# Intestinal IL-22RA1 signaling regulates *Chlamydia* deficient in plasmid-encoded pGP3 spreading to large intestine

**DOI:** 10.3389/fmicb.2025.1647731

**Published:** 2025-09-17

**Authors:** Qi Tian, Guangchi Fang, Jingyue Ma, Luying Wang, Zonghui Zuo, Tianyuan Zhang

**Affiliations:** ^1^Department of Obstetrics and Gynecology, Hunan Provincial Maternal and Child Health Care Hospital, Changsha, China; ^2^Tianjin Key Laboratory of Agricultural Animal Breeding and Healthy Husbandry, College of Animal Science and Veterinary Medicine, Tianjin Agricultural University, Tianjin, China; ^3^Department of Dermatovenereology, Tianjin Medical University General Hospital/Tianjin Institute of Sexually Transmitted Diseases, Tianjin, China; ^4^Department of Obstetrics and Gynecology, 3rd Xiangya Hospital, Central South University, Changsha, Hunan, China; ^5^Shanghai Institute of Virology, Shanghai Jiao Tong University School of Medicine, Shanghai, China; ^6^Key Lab of Molecular Virology and Immunology, Shanghai Institute of Immunity and Infection, Chinese Academy of Sciences, Shanghai, China

**Keywords:** *Chlamydia muridarum*, pGP3, IL-22, gastrointestinal tract, colonization

## Abstract

*Chlamydia trachomatis* is the most important infectious cause of tubal infertility and is frequently detected in the human gastrointestinal tract. *Chlamydia muridarum*, a murine pathogen, closely resembles the human pathogen *C. trachomatis*. Our previous studies showed that the pGP3-deficient *C. muridarum* mutant was restricted to the large intestine following intracolonic inoculation, suggesting that the pGP3-deficient mutant was killed by the tissue beyond the large intestine. Here, we report that the intra-ilenum, but not the intra-jejunum, to bypass the gastric barrier rescued the colonization of pGP3-deficient *C. muridarum*, suggesting that pGP3 is required to overcome host factors of the jejunum to help *C. muridarum* reach the colon. Moreover, mice genetically deficient in IL-22 not only rescued the colonization of pGP3-deficient *C. muridarum* following intrajejunal inoculation but also rescued the colonization of pGP3-deficient *C. muridarum* in the whole gastrointestinal tract tissues following intracolonic inoculation on day 14, suggesting a critical role of IL-22 in regulating chlamydial spread. Importantly, IL-22RA1 flox/flox and Villin-cre mice rescued the colonization of pGP3-deficient *C. muridarum* following intrajejunal inoculation, suggesting that intestinal epithelial-specific IL-22RA1 signaling is important for the spread of pGP3-deficient *C. muridarum* from the small intestine to the large intestine. These observations provide a platform for further research on intestinal IL-22RA1 signaling in regulating bacterial spread in the intestine. Therefore, host factors identified in the gastrointestinal tract may also contribute to the female lower genital tract barrier during sexually transmitted diseases.

## Highlights

- pGP3 is required for *C. muridarum* to spread large intestine following intrajejunal inoculation.
- IL-22 is an important factor for blocking the spread of pGP3-deficient *C. muridarum* from the small intestine to the large intestine.
- Intestinal epithelial IL-22RA1 signaling regulates *Chlamydia* organisms spreading from small intestine to large intestine.

## 1 Introduction

*Chlamydia trachomatis* (*C. trachomatis*) is a prevalent bacterial pathogen responsible for sexually transmitted infections (STIs) in humans (Budrys et al., 2012). Recent studies have demonstrated that *C. trachomatis* and *Chlamydia muridarum (C. muridarum)* colonize the gastrointestinal (GI) tract of their respective hosts (humans and mice) (Yang et al., 2014; Pospischil et al., 2009; Zhang et al., 2015; Craig et al., 2015; Peters et al., 2014; Gratrix et al., 2015; Musil et al., 2016; Gratrix et al., 2014). Notably, murine GI tract colonization by *C. muridarum* has been shown to influence both infection dynamics and pathogenicity in the genital tract, with these effects dependent on the sequence of exposure to the pathogen. However, the precise mechanisms underlying *C. trachomatis* colonization of the human gut remain poorly understood.

The murine model of *C. muridarum* infection has been used to study chlamydial pathogenesis and has revealed numerous chlamydial and host factors required for chlamydial induction or protection of hydrosalpinx (Tan et al., 2018; Wang et al., 2020; Wang et al., 2024; Wang et al., 2023). Chlamydial plasmid-encoded virulence factors essential for infection in the mouse genital tract have been shown to play equally critical roles in GI tract colonization (Zhong, 2018; Shao et al., 2017; Shao et al., 2017; Shao et al., 2017; Koprivsek et al., 2019). The absence of pGP3 in *Chlamydia* leads to two key deficiencies: attenuated genital tract infection and impaired gastrointestinal colonization (Liu et al., 2014; Lei et al., 2014; O'Connell et al., 2007; Chen et al., 2015). Our previous research revealed that pGP3, an outer membrane-associated protein, is crucial for *C. muridarum* to overcome the gastric barrier, thereby enabling persistent colonization in the colon (Zhang et al., 2019). Interestingly, when introduced via intracolonic inoculation, pGP3-deficient *Chlamydia* can still colonize the colon, suggesting that pGP3 triggers enhanced intestinal barrier defense, restricting bacterial dissemination.

Thus, investigating the mechanisms of *C. muridarum* interaction with the GI tract may enhance our understanding of *Chlamydia* pathogenesis. In the current study, we used a pGP3-deficient *Chlamydia muridarum-spreading* mouse model to determine the immunological basis of the intestinal barrier. We reported that mice genetically deficient in IL-22 rescued the colonization of pGP3-deficient *C. muridarum* following intrajejunal inoculation. Moreover, these mice also rescued the colonization of pGP3-deficient *C. muridarum* in the whole gastrointestinal tract following intracolonic inoculation on day 14. This suggests that IL-22 is an important factor for pGP3-deficient *C. muridarum* spread from the small intestine to the large intestine. Interestingly, IL-22RA1 flox/flox and Villin-cre mice rescued the colonization of pGP3-deficient *C. muridarum* following intrajejunal inoculation, suggesting that pGP3 is important for chlamydial evasion of intestinal epithelial-specific IL-22 expression. Thus, we revealed a novel method for selecting host factors from the GI tract, which may also contribute to the mouse genital tract.

## 2 Materials and methods

### 2.1 Chlamydial organisms

All *Chlamydia muridarum* clones used in this study were derived from the strain Nigg3 (Genbank accession number CP009760.1). The plasmid-free clone CMUT3G5 (GenBank accession# CP006974.1) was initially derived from Nigg3 (Lei et al., 2014), which was used for transformation with the plasmid pCM: GFP to create CM-pGFP (designated as wild type in the current study) or pCM: GFP with a premature stop codon in the *pgp3* gene to create CM-pGP3S (designated as mutant in the current study), as described previously (Liu et al., 2014; Liu et al., 2014). The genome and plasmid sequences of CM-pGFP and CM-pGP3S were nearly identical, except for a premature stop codon in *pgp3* in CM-pGP3S. As both were transformants, the plasmid copy numbers were similar. Both organisms were propagated in HeLa cells and purified as elementary bodies (EBs), as described previously (Zhang et al., 2015; Fan et al., 1998). Aliquots of the purified EBs were stored in SPG buffer (220 mM sucrose, 12.5 mM phosphate, and 4 mM L-glutamic acid, pH 7.5) at −80 °C until further use.

### 2.2 Mouse inoculation

Purified *C. muridarum* EBs were used to infect six-week-old C57BL/6 J mice (Shanghai Lingchang Biotechnology Co., Ltd) intra-jejunum, intra-ileum or intracolon with different inclusion-forming units (IFUs) as indicated in individual experiments.

IL-22 knockout mice were purchased from Cyagen: IL-22 knockout (KO; C57BL/6JCya-Il22em1/Cya, S-KO-10256) and Il22ra1-flox (Il22ra1-flox, S-CKO-07390). Villin-Cre mice were also obtained from Cyagen. The mice were inoculated with CM-pGFP (WT) or CM-pGP3S, as described below in the text.

Intracolonic inoculation was used to deliver 2 × 10^5^ live IFU organisms in 10 μL of SPG buffer to the mouse colon using an inoculation tube (NSET, catalog number 60010; ParaTechs Corp., Lexington, KY, United States).

Intrajejunal or intraileal inoculation: Mice were anesthetized using a mixture of isoflurane and oxygen. Once the mice were unconscious, their abdomens were shaved and sterilized with 70% ethanol. A small incision (approximately 0.25–0.5 in.) was made in the was created in the abdomen using a pair of scissors. The jejunum or ileum was partially pulled out of the body cavity using curved forceps. To the jejunum or ileum, 2 × 10^5^ IFUs of CM-pGP3S in 50 μL of SPG were inoculated using a 1-mL syringe (KDL, Shanghai, China). Care was taken not to remove excess intestines from the animal, completely pierce the intestine, or inject air bubbles. After the injection, the jejunum was placed back into the cavity, and the wound was closed using three to four surgical staples (#ACS- KIT, Braintree Scientific, Inc., Braintree, MA). The mice were resuscitated by placing them on a warm heating pad and supplying them with fresh air.

Note: For example, if the stock titer was 1 × 10^7^ IFU/μL, the stock was diluted 1:10 (10 μL + 90 μL SPG buffer) for a stock titer of 1 × 10^6^ IFU/μl. The stock was diluted 1:50 (20 μL + 980 μL SPG buffer) to a final stock titer of 2 × 10^4^ IFU/μL (2 × 10^5^ IFUs per 10 μL).

### 2.3 Titrating live chlamydial organisms recovered from swabs and tissue homogenates

To quantify live chlamydial organisms in rectal swabs, each swab was soaked in 0.5 mL of SPG, vortexed with glass beads, and the chlamydial organisms released into the supernatants were titrated on HeLa cell monolayers in duplicates. Infected cultures were processed for immunofluorescence assays as described previously (Tang et al., 2013). Inclusions were counted in five random fields per coverslip under a fluorescence microscope. Coverslips with fewer than one infectious unit (IFU) per field were counted. Coverslips exhibiting cytotoxicity in HeLa cells were excluded. The total number of IFUs per swab was calculated based on the mean number of IFUs per view, the ratio of the view area to that of the well, the dilution factor, and the inoculation volume. Where possible, the mean IFU/swab was derived from serially diluted duplicate samples for each swab. The total number of IFUs/swabs was converted to log10, which was used to calculate the mean and standard deviation across the mice in the same group at each time point.

To quantify live organisms in mouse organs and tissue segments, each organ or tissue segment was transferred to a tube containing 0.5–5 mL SPG, depending on the size of the organ. Each GI tract was cut into seven segments: stomach, duodenum, jejunum, ileum, cecum, colon, and anorectum (rectum). The organs and tissue segments were homogenized in cold SPG using a 2 mL tissue grinder (cat# K885300-0002, Fisher Scientific, Pittsburgh, PA, United States) or an automatic homogenizer (Omni Tissue Homogenizer, TH115; Kennesaw, GA, United States). The homogenates were briefly sonicated and centrifuged at 3,000 rpm for 5 min to pellet the remaining debris. The supernatants were titrated for live *C. muridarum* on HeLa cells, as described above. The results were expressed as log_10_ IFUs per organ or tissue segment weight.

### 2.4 Immunofluorescence assay

For immunofluorescence labeling of *C. muridarum* in HeLa cells, a rabbit antibody (R1604, raised with purified *C. muridarum* elementary bodies) was used as the primary antibody, which was visualized using goat anti-rabbit IgG conjugated to Cy2 (green, cat#111-225-144, Jackson ImmunoResearch Laboratories). The DNA dye Hoechst 3328 (blue, Sigma-Aldrich) was used to visualize the nuclei. Doubly labeled samples were used to count *C. muridarum* using a fluorescence microscope (MIX60, MshOt) with a CCD camera.

### 2.5 Counting inclusions and calculating IFUs

For each well, IFUs from five random views were counted under an objective lens using the appropriate magnification and were averaged. If one or fewer IFUs per view were found using a 10× objective lens, the entire well was considered. To determine the number of IFUs contained within the sample, the average number of IFUs per view derived from the five views was multiplied by the number of views possible in the total well per magnification, dilution, and factor reflecting the portion of the sample used for titration (Wang et al., 2025). After completing this procedure for each dilution in which IFUs were visible, the average number of IFUs was calculated and expressed as log10 transformed IFUs for statistical analyses.

### 2.6 Statistical analysis

The experimental data were analyzed using the Wilcoxon rank-sum test to compare the individual tissue types and overall large intestinal chlamydial burden. Fisher’s exact test was used to compare the frequencies of infection between the groups of mice.

## 3 Results

### 3.1 pGP3-deficient *Chlamydia muridarum* failed to spread to large intestine after direct delivery into jejunum

**Figure 1:**
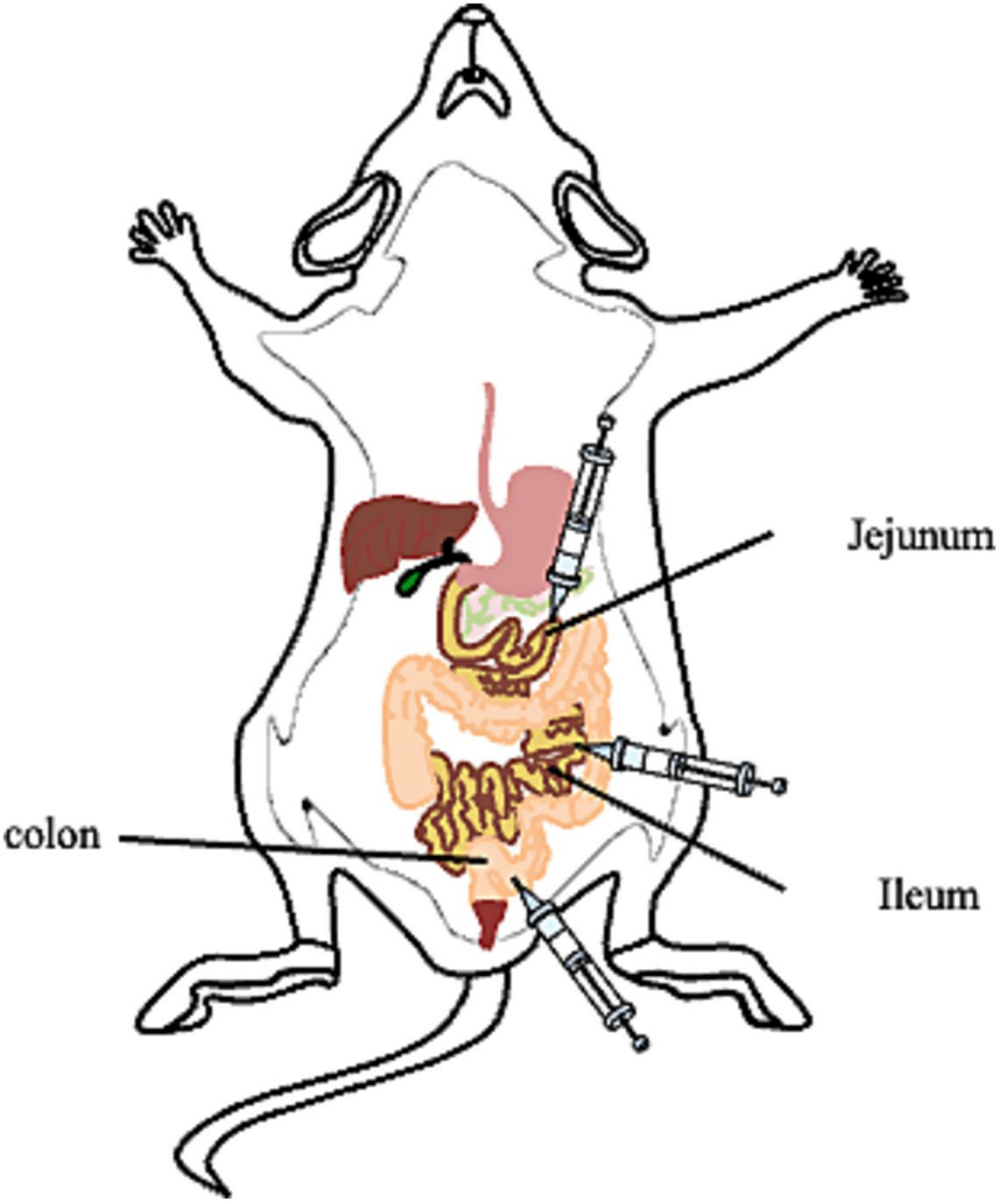
Photograph of the inoculation sites of the mice. Group of mice were inoculated with wild type *C. muridarum* (CM-pGFP) or pGP3-deficient *C. muridarum* (CM- pGP3S) following different inoculation sites.

**Figure 2:**
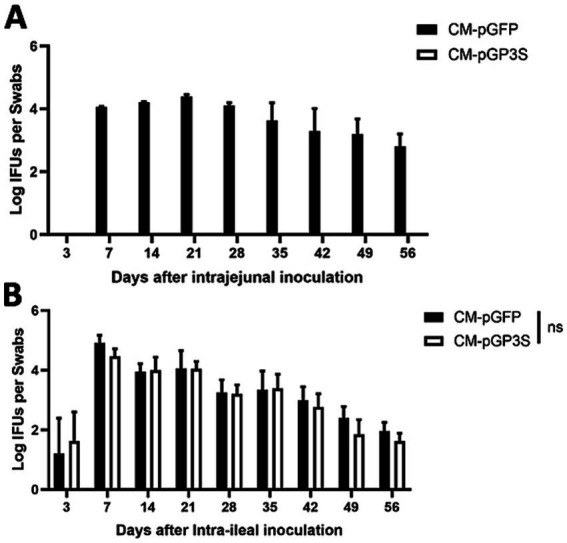
The pGP3-deficient *C. muridarum* mutant is able to colonize the gastrointestinal tract following an intraileal but not intrajejunal inoculation. Groups of C57BL/6J mice were inoculated with 2 × 10^5^ inclusion forming units (IFUs) of wild-type *C. muridarum* (CM-pGFP) or pGP3-deficient *C. muridarum* (CM-pGP3S) either intra-jejunum **(A)** or intra-ileum **(B)**. At different time points, as shown along the x-axis, mice were monitored for live chlamydial organism recovery from rectal swabs, and the results were expressed as Log10 IFUs per swab, as shown along the Y-axis. Each group consisted of 3–5 mice and data were obtained from 2 to 3 independent experiments.

We previously showed that the plasmid-encoded genital tract virulence factor pGP3 is essential for *C. muridarum* survival in the stomach of the GI tract (Zhang et al., 2019). To further define the organisms from which tissue sites are located beyond the large intestine to prevent *C. muridarum* from colonizing the large intestine, the different inoculation sites in the mice are shown in Figure 1, and we compared live organisms recovered from rectal swabs following intrajejunal versus intraileal inoculations (Figure 2). Following intrajejunal inoculation, no live organisms were recovered from mice inoculated with pGP3-deficient *C. muridarum*, although wild-type *C. muridarum* colonized the GI tract for the two-month period. However, when chlamydial organisms were directly inoculated into the ileum, the pGP3-deficient mutant was rescued and colonized the GI tract. Mice inoculated with the mutant continued to shed live organisms in rectal swabs throughout the two-month period. These results suggest that pGP3 is required for *C. muridarum* to spread in the large intestine following intrajejunal inoculation.

### 3.2 IL-22 is an important factor for blocking the spread of pGP3-deficient *Chlamydia muridarum* from the small intestine to the large intestine

Interleukin-22 (IL-22) is an important cytokine in the intestinal environment that is required to maintain intestinal homeostasis (Zenewicz et al., 2008; Gunasekera et al., 2020; Pavlidis et al., 2022; Huber et al., 2012; Ahlfors et al., 2014; Castleman et al., 2019; Mar et al., 2023). Intestinal tuft cells can also induce anti-Salmonella responses via NKp46 + ILC3 IL22 (Churchill et al., 2025). More importantly, it activates IL-22 production in ILCs to enhance host tissue defense following *C. difficile* infection (Mears et al., 2025). It is important to determine whether the host factor IL-22 is a key factor in regulating the spread of pGP3-deficient mutants to the large intestine after their direct delivery into the small intestine. Therefore, we compared chlamydial colonization in the GI tract of mice deficient in IL-22.

**Figure 3:**
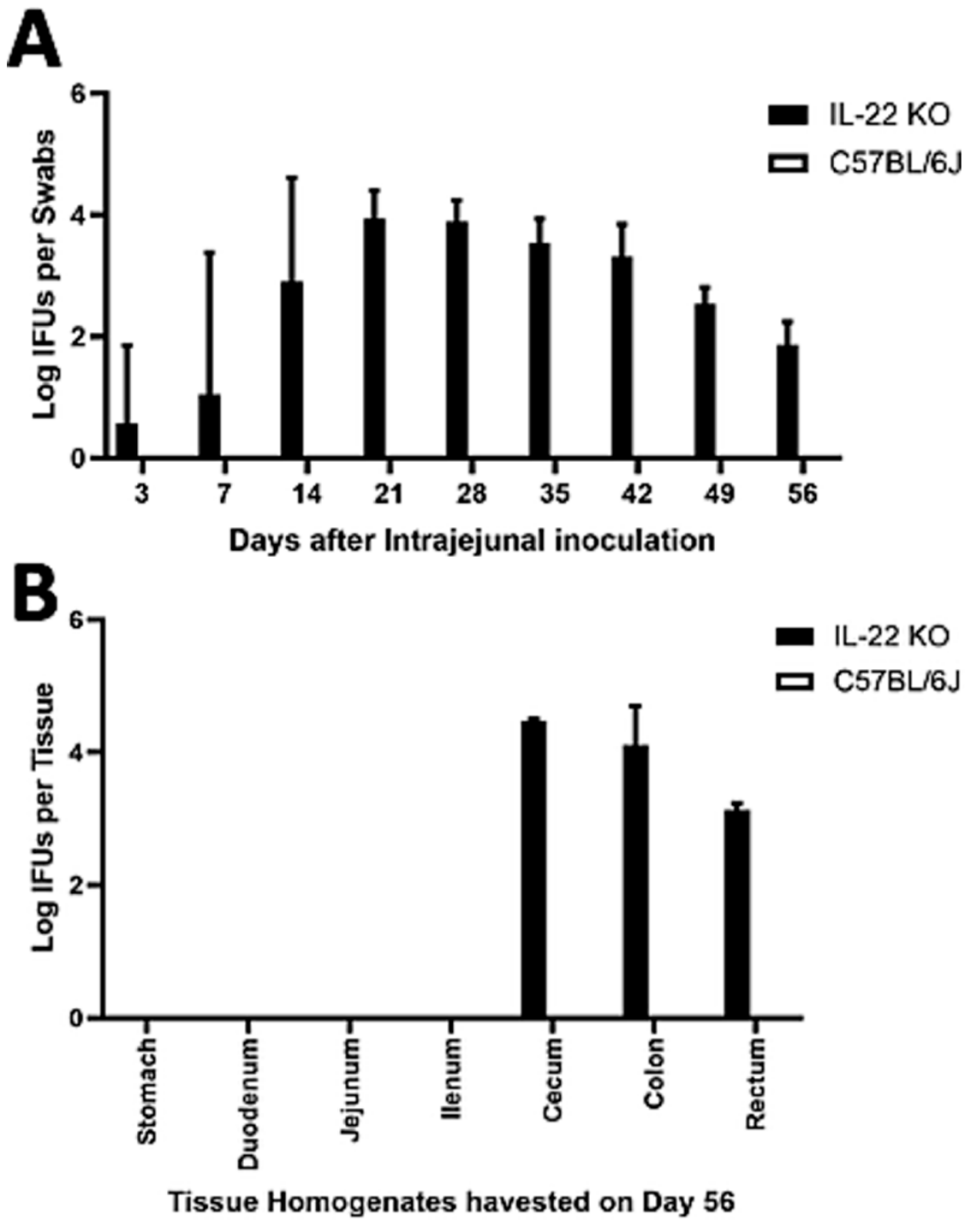
Deficiency in IL-22 rescued the pGP3-deficient *C. muridarum* mutant to colonize the gastrointestinal tract following an intra-jejunum inoculation. **(A)** Groups of mice deficient in Interleukin-22 (a key cytokine for regulating gut function, IL-22 KO) or without deficiency (wild-type C57BL/6J) were intra-jejunum inoculated with 2 × 10^5^ IFUs of pGP3-deficient *C. muridarum*. At different time points, as shown along the X-axis, mice were monitored for live chlamydial organisms in rectal swabs, and the results are expressed as Log_10_ IFUs per swab, as shown along the Y-axis. *N* = 5 mice in each group. Data were obtained from two independent experiments. **(B)** Parallel groups of mice with the same inoculation with pGP3-deficient mutant *C. muridarum* were sacrificed on day 56 after inoculation, as shown on top of each panel, for monitoring live organism recoveries from different gastrointestinal tissues from the stomach, duodenum to rectum, as indicated along the X-axis. Live organisms are expressed as Log10 IFUs per tissue, as shown along the Y-axis. Each group consisted of 3–5 mice, and the data were obtained from two independent experiments.

Following intrajejunal inoculation, the *C. muridarum* mutant colonized the GI tract of mice deficient in IL-22 (Figure 3A). By day 14 after inoculation, a significant number of live mutants were recovered from the rectal swabs of all IL-22 deficient mice. Overcoming host factor killing is necessary for the detection of intra-jejunum-inoculated chlamydial organisms in rectal swabs, and mice deficient in IL-22 can no longer produce the key host factor IL-22 in their small intestine. These observations suggest that IL-22 regulates the spread of *Chlamydia* from the small intestine to the large intestine. This conclusion is consistent with the observation that chlamydial mutant organisms were detected in the large intestines of IL-22-deficient mice for at least 56 days post-infection (Figure 3B).

**Figure 4:**
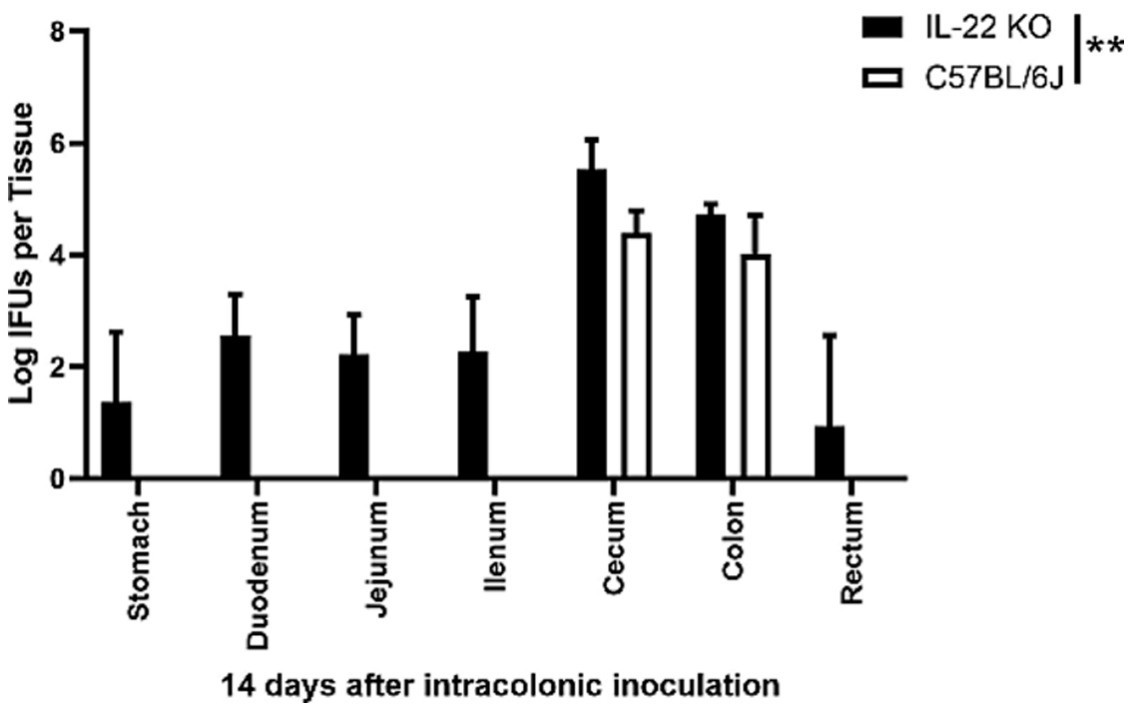
Intracolonically inoculated the pGP3-deficient *C. muridarum* is able to spread to extra-large intestine tissues in mice deficient in IL-22. Groups of mice deficient in Interleukin-22 or without deficiency (wild-type C57BL/6J) were inoculated with 2 × 10^5^ IFUs of pGP3-deficient *C. muridarum* intracolonically. Mice were sacrificed on day 14 after inoculation to monitor live organism recoveries from different gastrointestinal tissues from stomach, duodenum to rectum, as indicated along the X-axis. The live organisms were expressed as Log10 IFUs per tissue shown along the Y-axis. Each group has 3 to 5 mice and the data was obtained from 2 independent experiments.

We previously demonstrated that pGP3-deficient *C. muridarum* failed to spread to extra-large intestinal tissues in wild-type mice after intracolonic inoculation (Zhang et al., 2019). By day 14 after intracolonic inoculation in IL-22 deficient mice, the *C. muridarum* mutant was able to spread to the whole GI tract of mice deficient in IL-22 (Figure 4). This is consistent with the observation that mice deficient in IL-22 rescued pGP3-deficient *C. muridarum* colonization in the large intestine after direct delivery into the small intestine (Figure 3). Thus, we demonstrated that IL-22 is essential for preventing the spread of *Chlamydia* from the small intestine to the large intestine.

### 3.3 Intestinal epithelial specific IL-22RA1 deficient mice is sufficient for rescuing the spread of pGP3-deficient *C. muridarum* from small intestine to large intestine

**Figure 5:**
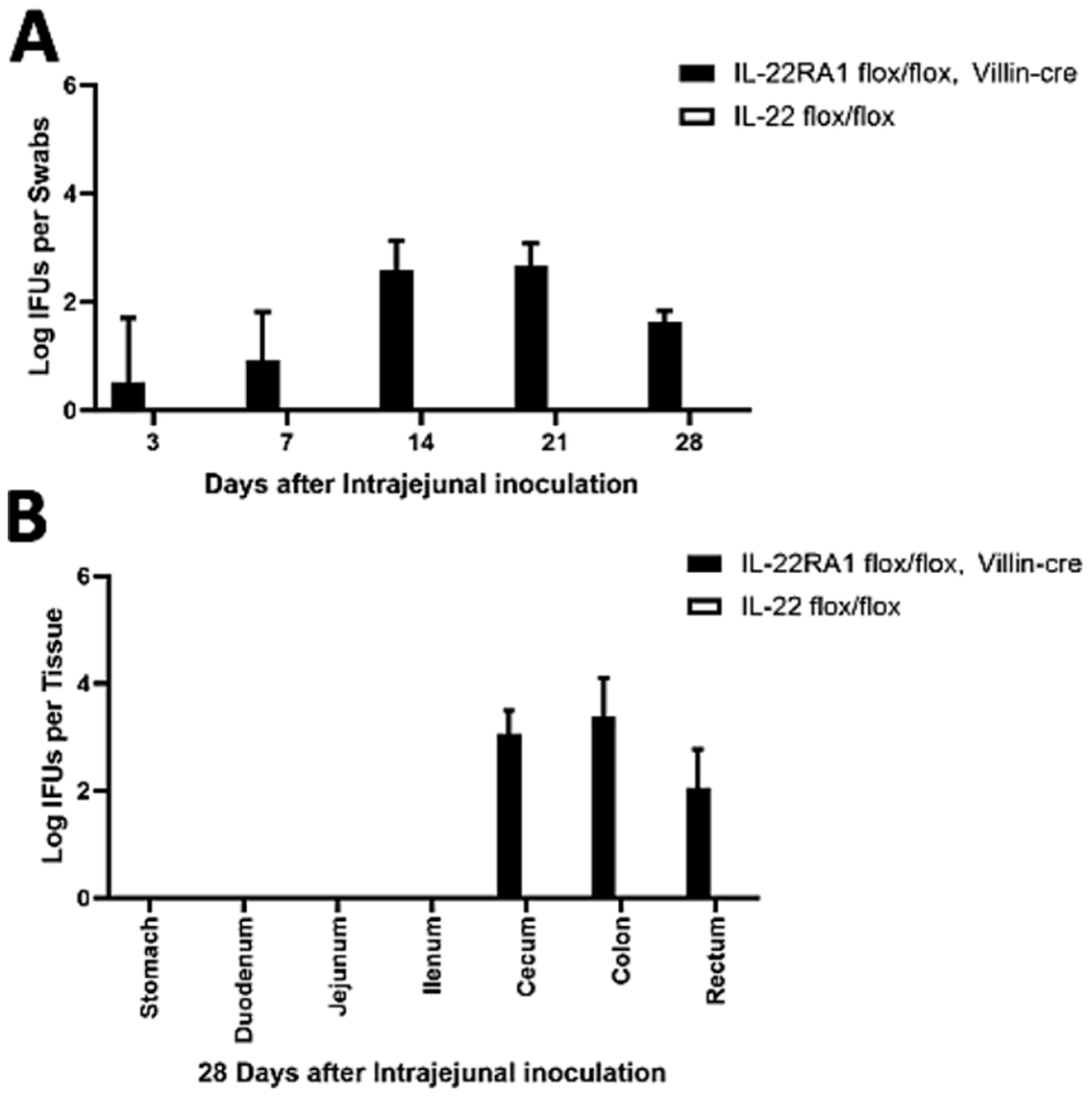
Intestinal epithelial-specific IL-22RA1 knockout mouse rescued the pGP3-deficient *C. muridarum* colonization following intra-jejunum inoculation. **(A)** Groups of Intestinal epithelial-specific IL-22RA1 knockout mice (IL-22RA1 flox/flox mice were bred with Villin-cre mice) or control (IL-22 flox/flox mice) were intra-jejunum inoculated with 2 × 10^5^ IFUs of pGP3-deficient *C. muridarum*. At different time points as shown along the X-axis, mice were monitored for live chlamydial organisms in rectal swabs and the results were expressed as Log_10_ IFUs per swab as shown along the Y-axis. *N* = 5 mice for each group. The data was obtained from 2 independent experiments. **(B)** Parallel groups of the same mice with the same inoculation with pGP3-deficient mutant *C. muridarum* were sacrificed on day 28 after inoculation, as shown on top of each panel, to monitor live organism recoveries from different gastrointestinal tissues from the stomach, duodenum to the rectum, as indicated along the X-axis. The live organisms were expressed as Log10 IFUs per tissue shown along the Y-axis. Each group has 3 to 5 mice and the data was obtained from 2 independent experiments.

To further determine whether intestinal IL-22RA1 signaling is necessary for blocking chlamydial spread, we detected chlamydial colonization in the GI tracts of intestinal epithelial-specific IL-22 knockout mice (IL-22ra1 flox/flox mice were bred with Villin-cre mice) (Figure 5). Mice inoculated with the mutant continued to shed live organisms in rectal swabs throughout the 1 month period (Figure 5A), and the pGP3-deficient mutant could spread to the large intestine (cecum and colon) (Figure 5B). These observations suggest that intestinal epithelial-specific IL-22RA1 signaling regulates the spread of *Chlamydia* from the small intestine to the large intestine.

## 4 Discussion

Although the obligate intracellular bacterium *C. trachomatis* is a human pathogen of the genital tract, it is frequently detected in the GI tract of mice. However, the significance and mechanisms of *C. trachomatis* colonization in the gut remain unknown. Since mouse-adapted *C. muridarum* colonizes the mouse GI tract (Rank and Yeruva, 2014; Dai et al., 2016; Perry and Hughes, 1999), when a naïve mouse is first exposed to *C. muridarum* in the GI tract, the mouse is orally immunized against subsequent chlamydial infections in the extra-gut tissues. Thus, investigating the mechanisms of *C. muridarum* interactions with the GI tract may promote our understanding of chlamydial pathogenic mechanisms and facilitate the development of oral vaccines against chlamydial infections in the genital tract. Therefore, murine models have been used to investigate the significance of *Chlamydia* in the GI tract of these animals.

These interesting models have motivated investigations of the mechanisms of *C. muridarum*-mouse gut interactions using various methods, such as *C. muridarum* mutants, knockout mice, or blockade. Using the failure of the CM-pGP3S mutant to spread from the small intestine to the large intestine, we determined that IL-22 and intestinal epithelial-specific IL-22RA1 signaling inhibited CM-pGP3S spread. First, IL-22 knockout mice showed significant spread of CMpGP3S from the small intestine to the large intestine following intrajejunal inoculation. Second, CM-pGP3S did not block the spread from the large intestine to the small intestine in IL-22 knockout mice following intracolonic inoculation. Thus, IL-22 may play a critical role in regulating the spread of bacteria into the large intestine. Finally, the intestinal epithelial-specific IL-22RA1 signaling regulation of chlamydial spreading correlated with intestinal epithelial-specific IL-22RA1 knockout mice rescued from CM-pGP3S spread following intrajejunal inoculation.

These observations led us to hypothesize that CM-pGP3S activates an immune response to block its spread to the large intestine. The current study revealed that intestinal epithelial IL-22RA1 signaling is a critical component of the CMpGP3S-activated barrier. However, the precise relationship between intestinal epithelial IL-22RA1 signaling and CM-pGP3S remains unclear. However, some questions remain unanswered. Which antimicrobial peptide production (CRAMP) by IL-22 or IL-22RA1 signal-mediated immunity is induced by CM-pGP3S? Which microbiome-mediated immunity blocks CMpGP3S spread via IL-22 or IL-22RA1 signals?

Although, knowledge gained from mouse models may not be applicable to *C. trachomatis* infections in humans. Nevertheless, the mechanistic information obtained from *C. muridarum* interactions with mouse mucosal tissues may still be informative for understanding how *C. trachomatis* interacts with human mucosal tissues in the genital tract (Zhao et al., 2015). It is worth noting that the focus of the current study was on *C. muridarum* spread along the mouse gut. Clearly, more mechanisms are required to investigate the spread of chlamydial organisms from the small intestine to the large intestine.

## 5 Statistics analyses

The Wilcoxon rank-sum test was used to compare the number of live organisms in the IFUs between different samples.

## Data Availability

The original contributions presented in the study are included in the article/supplementary material, further inquiries can be directed to the corresponding authors.
